# Real-Time Attitude Estimation for Spinning Projectiles by Magnetometer Based on an Adaptive Extended Kalman Filter

**DOI:** 10.3390/mi14112000

**Published:** 2023-10-28

**Authors:** Ge Zhang, Xiaoming Zhang, Lizhen Gao, Jun Liu, Jie Zhou

**Affiliations:** 1 Key Laboratory of Instrument Science and Dynamic Testing Ministry of Education, North University of China, Taiyuan 030051, China; gezhang_auto@163.com (G.Z.); liuj@nuc.edu.cn (J.L.); zj13593199734@163.com (J.Z.); 2 Key Laboratory of Quantum Sensing and Precision Measurement, Taiyuan 030051, China; 3 Institute of Instrument and Electronics, North University of China, Taiyuan 030051, China; 4 School of Electrical and Control Engineering, North University of China, Taiyuan 030051, China; gaolzh@163.com

**Keywords:** spinning projectile, geomagnetic information, AEKF, attitude estimation

## Abstract

The attitude measurement system based on geomagnetic information offers advantages such as small space requirements, fast response times, excellent resistance to high-overload conditions, and cost-effectiveness. However, during the flight process of a high-mobility guided spinning projectile, calculating attitude based on geomagnetic information often leads to non-unique solutions. To address this challenge, this paper proposes the Adaptive Extended Kalman Filter (AEKF) attitude estimation algorithm, based on geomagnetic vector information. Based on the analysis of the short-term attitude motion characteristics of the projectile, the Kalman state system equation and the nonlinear observation equation are established, along with real-time correction of the yaw angle and adaptive updates of parameters. The effectiveness of the algorithm is verified by simulations and experiments, demonstrating its ability to eliminate the dual solution problem inherent in traditional Single-Epoch algorithms. It notably improves the accuracy of pitch and roll angle estimation while providing precise estimates of attitude angular rates. Furthermore, the algorithm effectively mitigates the impact of magnetic disturbances on attitude determination. The proposed method has many potential applications in attitude measurement and navigation using geomagnetic data.

## 1. Introduction

Accurately measuring the attitude angles and angular rates of spinning projectiles is a fundamental requirement for effective projectile control. Precise acquisition of the projectile’s attitude is vital for the guided ammunition’s control system to ensure accurate target engagement [1]. On the other hand, determining the attitude enables incorporating a damping circuit into the projectile control system, enhancing its dynamic performance [2,3]. Consequently, obtaining accurate attitude measurements of spinning projectiles is of utmost significance for guided ammunition applications.

In order to mitigate the impact of aerodynamic asymmetry, mass eccentricity, and assembly errors on flight stability, most projectiles adopt a high-speed rotating mechanism [4,5]. In in-flight environments with high axial velocities and intense overloads, commonly employed MEMS gyroscopes encounter challenges such as saturation, performance degradation, failure subsequent to overloading, difficulty in initial alignment, and so on [6,7]. Furthermore, its performance degradation is irreversible, which reduces its ability to provide the accurate data required for the solution process.

In recent years, geomagnetic information has been increasingly used to determine the attitude of spinning projectiles. Attitude measurement systems based on geomagnetism offer advantages such as small space occupation, fast response speed, good anti-high overload performance, no accumulated error over time, low cost, and so on [8,9]. Most significantly, the attitude can be determined through geomagnetic vector measurements, even in high overload and dynamic flight environments. Meanwhile, as has been validated, the magnetic disturbance imposed on the measurement of the magnetometer often causes a significant decrease in inclination estimation accuracy [10].

Many effective methods were proposed to solve the problem of real-time attitude estimated by magnetic measurement information. Deng [11] employed the Phase-Locked Loop (PLL) technique to track the phase of the radial magnetometer signal, thus obtaining the roll angle of the flying body. Its estimation accuracy is relatively low, and it cannot acquire pitch and yaw information. The Triaxial Magnetometer Extremum Ratio method [12,13,14] is closely tied to the measurement accuracy of the triaxial magnetometer. Therefore, it is essential to utilize online error correction technology [15,16] to ensure the magnetometer’s measurement accuracy, albeit at the cost of increasing system complexity. STFT (short-time Fourier transform) adopts the Fourier transform to estimate angular rate well [17,18], but there is a contradiction between the accuracy and real-time performance of this method in assessing attitude, limiting its application in highly dynamic environments. Differential filtering uses the difference in magnetic measurement signals to obtain angular velocity information [19]. However, the error obtained is significant, especially when there is a substantial measurement error in the magnetic signal. The angular rate information is obtained by measuring the time of each zero crossing with a sinusoidal magnetic signal in the zero-crossing detection method [2,20]. Unfortunately, the certainty of the data update rate is compromised by the direct correlation: the faster the axial speed, the quicker the data update rate becomes. Roberts [21] utilized the triaxial magnetometer output vector and layout direction vector to ascertain the attitude of the flying body for high-speed rotating flying bodies, such as spinning projectiles, in highly dynamic environments. Ensuring the accuracy of vector observations can be challenging. The dynamics model of the flying body can also be used to determine attitude information [4,10]. However, the force and torque applied to spinning projectiles are very complex because of their high speed and varied temperature, making an accurate dynamic model challenging to establish. Kalman filter-based projectile attitude estimation methods [22,23] require combining onboard sensors such as gyroscopes, accelerometers, and magnetometers to determine attitude. In high-overload environments, these algorithms face the inevitable and irreversible issue of performance degradation in sensors such as gyroscopes, and their cost and complexity are also critical concerns.

In this article, the geomagnetic measurement method based on the Extended Kalman Filter (AEKF) is proposed to overcome the limitations of existing methods for the attitude measurement of a spinning projectile. This method eliminates the dual solution problem inherent in traditional Single-Epoch algorithms for attitude angle determination while simultaneously providing attitude angular rate information. Based on the short-term attitude and motion characteristics of the projectile, a quadratic polynomial model for pitch angle and roll angle with respect to time is established. Attitude and its conversion rates are selected as states, and real-time correction of the yaw angle is incorporated to formulate the state system equation. Furthermore, a nonlinear observation equation is established based on the direction cosine attitude transformation relationship of the geomagnetic vector in both the launch and projectile coordinate systems. Experimental results suggest that the proposed method effectively reduces real-time measurement errors in the pitch and roll angles of spinning projectiles and mitigates the impact of magnetic disturbance on attitude determination.

## 2. Materials and Methods

### 2.1. Magnetic Attitude Matrix

For the convenience of analysis, suppose the projectile is in external ballistic flight, and the geomagnetic field vector in the launch coordinate system (called ‘*f*’ system in the following) at the measuring point of the magnetic sensor on the projectile is $H^{f}={\left[\begin{array}{ccc} H_{x}^{f} & H_{y}^{f} & H_{z}^{f} \end{array}\right]}^{T}$. According to the conversion relationship from the launch coordinate system to the projectile coordinate system (called the ‘*b*’ system in the following), the projection of the geomagnetic field vector in the projectile coordinate system satisfies:

(1) $$H^{b}=C_{f}^{b}H^{f}$$

where $C_{f}^{b}$ is the attitude transformation matrix.

The transformation relationship from the launch coordinate system *Oxyz* to the projectile coordinate system *Ox_b_y_b_z_b_* follows the Euler angle rotation sequence of ‘*ψ*-*θ*-*γ*’. The matrix $C_{f}^{b}$ can be expressed as:

(2) $$C_{f}^{b}=R_{x}(\gamma )R_{z}(\theta )R_{y}(\psi )=\left[\begin{array}{ccc} \cos\psi \cos\theta & \sin\theta & -\sin\psi \cos\theta \\ \sin\gamma \sin\psi -\cos\gamma \cos\psi \sin\theta & \cos\gamma \cos\theta & \sin\gamma \cos\psi +\cos\gamma \sin\psi \sin\theta \\ \cos\gamma \sin\psi +\sin\gamma \cos\psi \sin\theta & -\sin\gamma \cos\theta & \cos\gamma \cos\psi -\sin\gamma \sin\psi \sin\theta \end{array}\right]$$

where *ψ* is yaw angle, *θ* is pitch angle, and *γ* is roll angle.

### 2.2. Traditional Single-Epoch Attitude Algorithm

According to the analysis above, the rotating ammunition has a high initial and axial rotational angular velocity after being launched from the barrel. In the initial uncontrolled flight phase of the outer ballistic trajectory, the gyro-fixed axis effect caused by the high rotation of the projectile axis makes the direction of the projectile stable in the launch coordinate system. At this stage, the yaw angle *ψ* of the projectile remains unchanged, approximately zero. Due to the spatial distribution of the main geomagnetic field and geomagnetic anomaly field, there is little change in the scale of tens of kilometers. For short- and medium-range rotating ammunition, the geomagnetic field vector can be regarded as a constant vector during outer ballistic flight, which can be obtained before the ammunition is launched. For long-range ammunition, the geomagnetic field vector at the moving point of the projectile can be calculated in real time from the geomagnetic model based on the three-dimensional position information of the projectile.

Considering that the flight trajectory of the projectile is roughly in the launch plane, the yaw angle of the projectile body can be assumed to be zero. Thus, the attitude transformation matrix (1) can be simplified as:

(3) $$C_{f}^{b}\approx R_{x}(\gamma )R_{z}(\theta )R_{y}(0)=\left[\begin{array}{ccc} \cos\theta & \sin\theta & 0\\ -\cos\gamma \sin\theta & \cos\gamma \cos\theta & \sin\gamma \\ \sin\gamma \sin\theta & -\sin\gamma \cos\theta & \cos\gamma \end{array}\right]$$

According to the linearization approximation theory of nonlinear system, the three components in the projectile coordinate system can be expressed as:

(4) $$\begin{array}{l} H_{x}^{b}=H_{x}^{f}\cos\theta +H_{y}^{f}\sin\theta \\ H_{y}^{b}=-H_{x}^{f}\cos\gamma \sin\theta +H_{y}^{f}\cos\gamma \cos\theta +H_{z}^{f}\sin\gamma \\ H_{z}^{b}=H_{x}^{f}\sin\gamma \sin\theta -H_{y}^{f}\sin\gamma \cos\theta +H_{z}^{f}\cos\gamma \end{array}$$

According to the universal equation of the trigonometric function:

(5) $$\sin\theta =\frac{2\tan\left(\theta /2\right)}{1+\tan^{2}\left(\theta /2\right)};\cos\theta =\frac{1-\tan^{2}\left(\theta /2\right)}{1+\tan^{2}\left(\theta /2\right)}$$

Then,

(6) $$\theta =2\arctan\left(\frac{H_{y}^{f}\pm \sqrt{{\left(H_{x}^{f}\right)}^{2}+{\left(H_{y}^{f}\right)}^{2}-{\left(H_{x}^{b}\right)}^{2}}}{H_{x}^{b}+H_{x}^{f}}\right)$$

It can be seen from Equation (6) that there is a dual solution problem for the pitch angle. Through the analysis of the equation, it can be concluded that the problem occurs when the value ${\left(H_{x}^{f}\right)}^{2}+{\left(H_{y}^{f}\right)}^{2}-{\left(H_{x}^{b}\right)}^{2}$ goes through 0. In this case, its physical significance can be explained as the straight line where the longitudinal axis of the projectile passes through the projection of the geomagnetic vector on the emission plane. The results of the roll angle have the same problem as shown in Equation (8).

(7) $$\tan\gamma =\frac{H_{y}^{b}H_{z}^{f}+H_{z}^{b}\left(H_{x}^{f}\sin\theta -H_{y}^{f}\cos\theta \right)}{H_{z}^{b}H_{z}^{f}-H_{y}^{b}\left(H_{x}^{f}\sin\theta -H_{y}^{f}\cos\theta \right)}$$

(8) $$\gamma =\arctan\left[\frac{H_{y}^{b}H_{z}^{f}+H_{z}^{b}\left(H_{x}^{f}\sin\theta -H_{y}^{f}\cos\theta \right)}{H_{z}^{b}H_{z}^{f}-H_{y}^{b}\left(H_{x}^{f}\sin\theta -H_{y}^{f}\cos\theta \right)}\right]$$

According to Equations (7) and (8), the solution of the roll angle is related to the pitch angle. Therefore, accurately determining the sign of the pitch angle is the basis for accurately solving the roll angle.

To address the issue of pitch angle ambiguity, the conventional Single-Epoch attitude determination method relies on the relationship between the angle (*ε*) formed by the projection of the geomagnetic vector onto the launch plane and the longitudinal axis (X-axis) of the projectile’s coordinate system, along with the initial value of the pitch angle (*θ*_0_) and the change in pitch angle (*θ*). Initially, during the launch phase, the value of the pitch angle is determined by comparing *θ*_0_ and *ε*. If the initial pitch angle *θ*_0_ ≥ *ε*, the larger of the two values is selected. During the subsequent flight, as the pitch angle decreases to a value less than ε, the smaller of the two values is chosen, and vice versa.

However, the Single-Epoch attitude determination method relies on current sensor measurements without incorporating historical data, rendering it incapable of making informed decisions regarding the pitch angle’s ambiguity based on its motion characteristics. In high-dynamic and adverse operational environments, the high rotational speed of the projectile can introduce considerable noise, leading to erroneous pitch angle determinations. Consequently, incorrect calculations may yield inaccurate roll angle readings, significantly impacting the projectile’s spin control.

### 2.3. The Adaptive Extended Kalman Filter (AEKF) Attitude Estimation Algorithm

Due to the inevitable influence of magnetic disturbances on geomagnetic information, the AEKF in this article can prevent the issues of divergence or insufficient accuracy that are commonly encountered in algorithms based on Kalman filtering, such as the EKF.

Based on the continuous smoothness of the projectile’s attitude change, assuming that the pitch and roll angles are quadratic polynomials within a short period of time, the pitch and roll angles can be expressed as:

(9) $$\left\{ \begin{array}{l} \theta =a_{\theta }t^{2}+b_{\theta }t+c_{\theta }\\ \gamma =a_{\gamma }t^{2}+b_{\gamma }t+c_{\gamma } \end{array}\right.$$

where *a_i_, b_i_, c_i_* (*i* = *θ*, *γ*) are the quadratic, linear, and coefficients of the respective attitude angle changes.

In order to maximize the provision of attitude information by the Adaptive Extended Kalman Filter (AEKF), the correction of the yaw angle’s influence on the geomagnetic three components (*H^f^*) in the body coordinate system is performed. The pitch and roll angles, along with their first- and second-order rate derivatives, have been designated as the states of the AEKF. Consequently, the state vector is delineated as follows:

(10) $$X={\left[\begin{array}{cccccc} \theta & \dot{\theta } & \ddot{\theta } & \gamma & \dot{\gamma } & \ddot{\gamma } \end{array}\right]}^{T}$$

where *θ* is the pitch angle and *γ* is the roll angle.

The state model of the filter can be derived from Equation (9) as presented below:

(11) $$\dot{X}(t)=\Phi X(t)+G(t)w(t)$$

with the following definitions:

(12) $$\Phi (t)=\left[\begin{array}{cc} \Phi_{\theta } & 0_{3\times 3}\\ 0_{3\times 3} & \Phi_{\gamma } \end{array}\right],\Phi_{\theta }=\Phi_{\gamma }=\left[\begin{array}{ccc} 0 & 1 & 0\\ 0 & 0 & 1\\ 0 & 0 & 0 \end{array}\right]$$

(13) $$G(t)=\left[\begin{array}{c} G_{\theta }\\ G_{\gamma } \end{array}\right],G_{\theta }=\left[\begin{array}{cc} 0 & 0\\ 0 & 0\\ 1 & 0 \end{array}\right],G_{\gamma }=\left[\begin{array}{cc} 0 & 0\\ 0 & 0\\ 0 & 1 \end{array}\right]$$

(14) $$w(t)={\left[w_{\varepsilon x},w_{\varepsilon y},w_{\varepsilon z},w_{\zeta x},w_{\zeta y},w_{\zeta z}\right]}^{T}$$

where *w_εx_*, *w_εy_*, *w_εz_* are the noise term of the pitch angle; *w_ζx_*, *w_ζy_*, *w_ζz_* denote the noise term of the roll angle.

Suppose *w(t)* is a zero-mean Gaussian white noise vector. It satisfies the following conditions: E[*w*(*t*)] = 0, E[*w(t) wT*(*τ*)] = *q*(*t*)*δ*(*t* − *τ*), *q*(*t*) is the variance intensity matrix of white noise.

According to the discretization theory of a continuous-time linear system, the equivalent discretized form of the continuous state equation is:

(15) $$X_{k}=\Phi_{k/k-1}X_{k-1}+\eta_{k-1}$$

where *X_k_* = *X*(*t_k_*).

Based on the direction cosine attitude transformation relationship of the geomagnetic vector between the launch coordinate system and the projectile body coordinate system, the corresponding filtering measurement model can be deduced. As a result, the discretization equation is formulated as follows:

(16) $$H_{k}^{b}=f\left[X_{k}\right]+n_{k}$$

where *n_k_* is a Gaussian white noise vector whose average value is zero, independent of noise *η_k_*, and satisfies the following conditions: E[*n_k_*] = 0, E[*n_k_n_j_^T^*] = *R**_k_**δ*(*k* − *j*), where *R**_k_*** is the variance matrix of the white noise of the magnetometer.

According to the linearization approximation theory of nonlinear systems, the nonlinear equations are Taylor-expanded around the nominal operating point *X_k_,* with higher-order terms neglected and only the linear terms retained. This process yields the filtering equation related to state deviations. Following the Extended Kalman Filter (EKF) methodology, the nonlinear measurement equation is linearized as follows:

(17) $$J_{k}=\frac{\partial H^{b}}{\partial X_{k}}=\left[\begin{array}{cccccc} {\frac{\partial H^{b}}{\partial \theta }|}_{X_{k}} & 0_{3\times 1} & 0_{3\times 1} & {\frac{\partial H^{b}}{\partial \gamma }|}_{X_{k}} & 0_{3\times 1} & 0_{3\times 1} \end{array}\right]$$

with the following definitions:

(18) $$\frac{\partial H^{b}}{\partial \theta }=\left[\begin{array}{ccc} -\sin\theta & \cos\theta & 0\\ -\cos\gamma \cos\theta & -\cos\gamma \sin\theta & 0\\ \sin\gamma \cos\theta & \sin\gamma \sin\theta & 0 \end{array}\right]\left[\begin{array}{c} H_{x}^{f}\\ H_{y}^{f}\\ H_{z}^{f} \end{array}\right]$$

(19) $$\frac{\partial H^{b}}{\partial \gamma }=\left[\begin{array}{ccc} 0 & 0 & 0\\ \sin\gamma \sin\theta & -\sin\gamma \cos\theta & \cos\gamma \\ \cos\gamma \sin\theta & -\cos\gamma \cos\theta & -\sin\gamma \end{array}\right]\left[\begin{array}{c} H_{x}^{f}\\ H_{y}^{f}\\ H_{z}^{f} \end{array}\right]$$

Upon transforming the filtering equation pertaining to state deviations, the recursive filtering equation for the nonlinear system using the state *X_k_* as the estimator can be derived as follows:

(20) $$\begin{array}{l} {\hat{X}}_{k/k-1}=\Phi_{k/k-1}{\hat{X}}_{k-1}\\ P_{k/k-1}=\Phi_{k/k-1}P_{k-1}\Phi_{k/k-1}^{T}+\Gamma_{k-1}Q_{k-1}\Gamma_{k-1}^{T}\\ K_{k}=P_{k/k-1}J_{k}^{T}{\left(J_{k}P_{k/k-1}J_{k}^{T}+R_{k}\right)}^{-1}\\ {\hat{X}}_{k}={\hat{X}}_{k/k-1}+K_{k}\left(H_{k}^{b}-C_{f}^{b}H_{k}^{f}\right)\\ P_{k}=\left(I-K_{k}J_{k}\right)P_{k/k-1} \end{array}$$

The adaptive process is as follows:

(21) $$\begin{array}{l} d_{k}=(1-\lambda )(1-\lambda^{k})\\ \nu_{k}=H_{k}^{b}-C_{f}^{b}H_{k}^{f}\\ R_{k}=(1-d_{k})R_{k}+d_{k}((I-J_{k}K_{k})\nu_{k}\nu_{k}{}^{T}{\left(I-J_{k}K_{k}\right)}^{T}+J_{k}P_{k/k-1}J_{k}{}^{T})\\ Q_{k}=\left(1-d_{k}\right)Q_{k-1}+d_{k}\left(K_{k}\;v_{k}v_{k}{}^{T}K_{k}{}^{T}\right) \end{array}$$

where *λ* represents the forgetting factor, 0 < *λ* < 1. When calculating the predicted state covariance matrix, Equation (21) continuously estimates and corrects the covariance matrix of system noise in real time. This reduces the state estimation error, enabling the AEKF attitude estimation process for the spinning projectile.

The initial values *X*_0_ and *P*_0_ of the AEKF filter can be determined using the attitude solution from a Single-Epoch geomagnetic attitude measurement algorithm and launch parameters. The noise covariance matrix *Q* of the filtering system can be obtained based on prior knowledge of the projectile’s motion law. The measurement noise covariance matrix *R* is estimated and determined based on the compensation accuracy of the magnetic sensor.

## 3. Results and Discussion

### 3.1. Simulation Experiments

**Figure 1:**
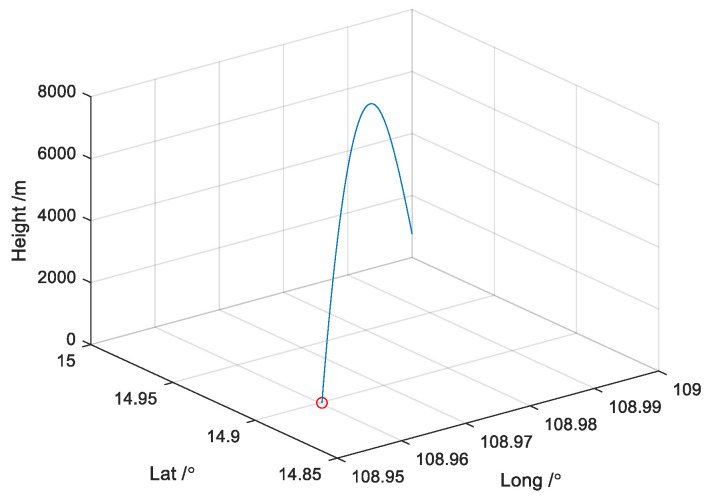
The trajectory of flight in simulation.

**Figure 2:**
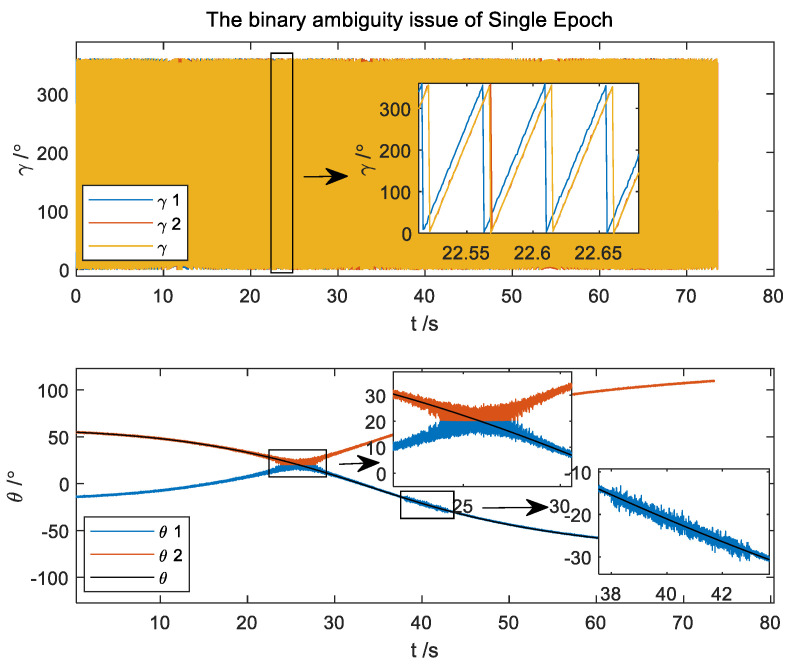
The binary ambiguity issue of the Single-Epoch attitude algorithm.

Based on the motion characteristics during flight, we made the assumption that the yaw angle of the projectile remains unchanged, approximately zero. The pitch angle gradually decreases due to the influence of gravity, and the roll angle changes rapidly. The roll angle speed follows an exponential decay law and obeys the modified Rouge empirical formula, which is assumed as follows:

(22) $$\dot{\gamma }=\omega_{x}^{b}=\omega_{0}\exp\left(-0.4k\frac{LD^{3}}{A}t\right)$$

where *ω*_0_ is the equivalent rotational angular rate of the muzzle of the rotating ammunition, *L* is the length of the projectile, *D* is the diameter of the projectile, *A* is the polar moment of inertia of the projectile, *k* is the correction coefficient, according to the analysis above, the trajectory is designed as depicted in Figure 1:

The results using the traditional Single-Epoch algorithm are shown in Figure 2. There is magnetic interference around 40 s. A binary ambiguity issue is presented in the pitch and roll angles obtained by the Single-Epoch attitude method.

**Figure 3:**
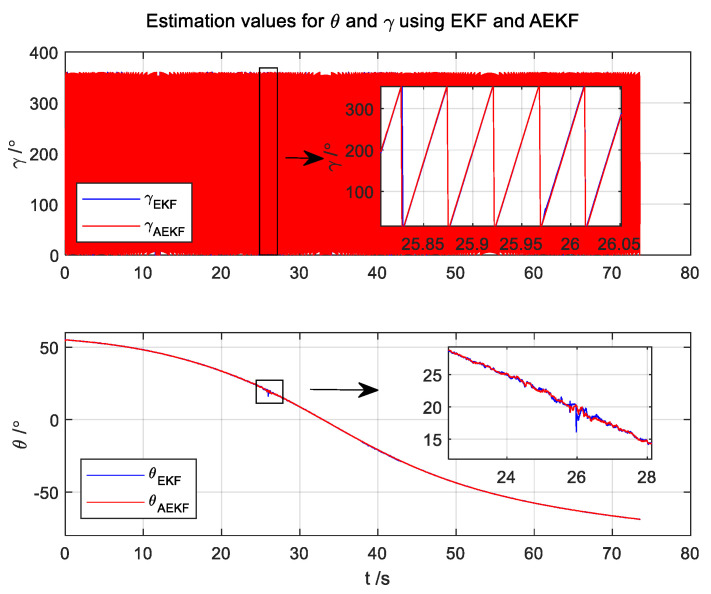
The simulation results are based on the presented algorithm.

The binary ambiguity issue of the traditional method is eliminated by the AEKF attitude algorithm, as depicted in Figure 3.

**Figure 4:**
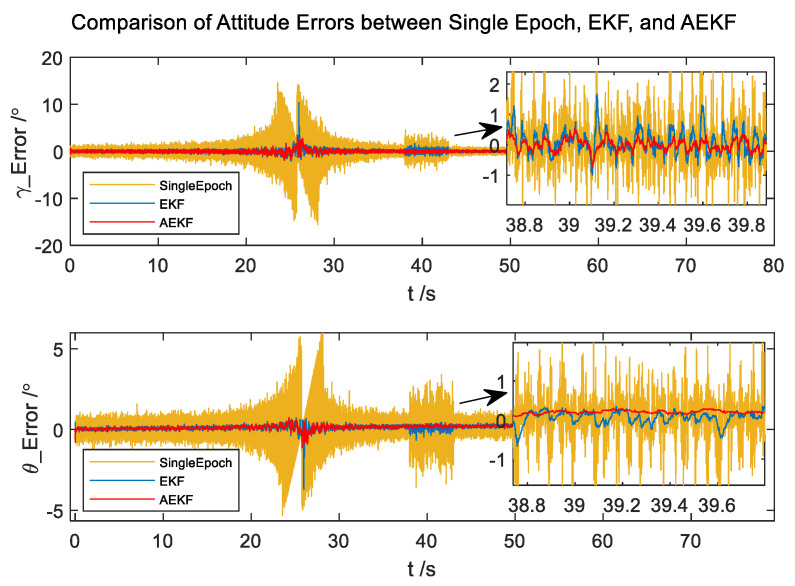
The Comparison of Attitude Errors between Single-Epoch, EKF, and AEKF.

The comparison between the attitude errors derived from the Single-Epoch, EKF, and AEKF is shown in Figure 4. The standard deviations of roll and pitch angles are presented in Table 1.

It can be seen from Figure 4 that the presented method, as compared to the traditional Single-Epoch method and EKF, does not exhibit binary ambiguity issues and offers significantly higher precision. Due to the influence of the aerodynamic force on the trajectory during flight, the yaw angle changed by 0.3° in the process. However, the algorithm considers historical data during the estimation process, keeping the maximum errors of pitch within 1°and roll angles within 1.5°, ensuring the continual validity of estimations. Compared with the error of the angles obtained by the Single-Epoch method, the standard deviation of roll angle error is reduced by four times. Similarly, the pitch angle standard deviation decreases four times throughout the process and 14 times in magnetic disturbance regions. Furthermore, it can be observed that the standard deviation of attitude angle errors obtained from the AEKF algorithm is smaller than that obtained from the EKF algorithm in the entire process and demonstrates superior adaptability compared to EKF, resulting in a twofold improvement in attitude accuracy in regions with magnetic disturbance. The convergence process can be accomplished within 100 milliseconds, showcasing the algorithm’s characteristics of high precision and real-time capability.

**Figure 5:**
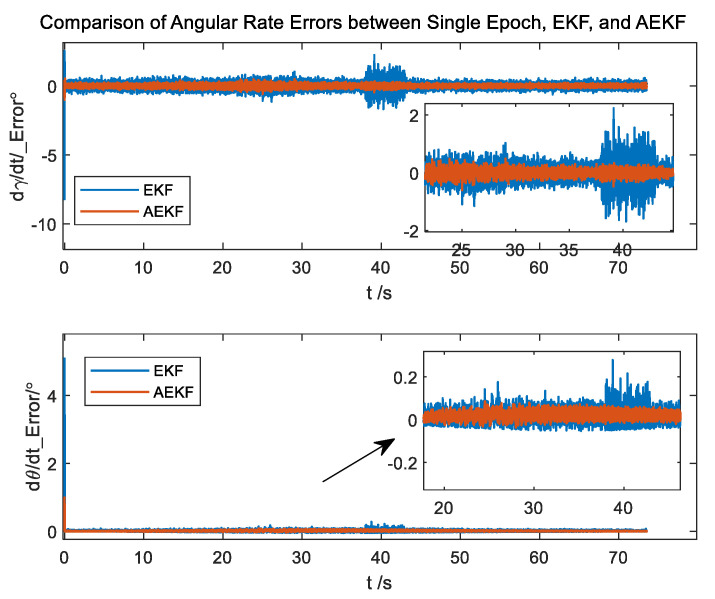
The errors of the angular rate.

More importantly, the AEKF can provide the first-order and second-order rate of change for both pitch and roll angles, with the first-order rate represented as the angular rate depicted in Figure 5. The standard deviation of attitude angular rate errors is presented in Table 2. This capability is crucial for controlling the attitude of a spinning projectile but cannot be estimated using conventional methods.

It can be observed that the standard deviation of attitude angular rate errors of the AEKF algorithm is smaller than that obtained from the EKF algorithm in the entire process and decreases three times in magnetic disturbance regions. This demonstrates the effectiveness of the algorithm in estimating angular rates.

### 3.2. Semi-Physical Experiments

**Figure 6:**
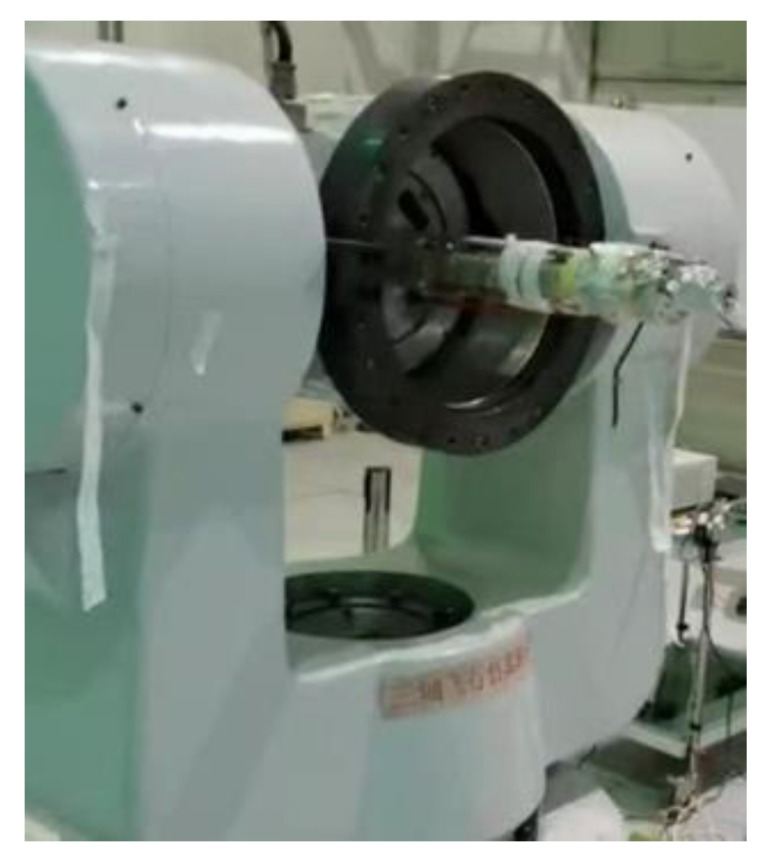
Three-axis high-precision flight simulation turntable.

In order to test the actual performance of the proposed algorithm based on AEKF, semi-physical experiments were conducted with a magnetic attitude measurement system, which consists of a triaxial magnetometer (HMC1053) produced by Honeywell and an STM32 single-chip microcomputer. The magnetic measurement system was fixed on the three-axis high-precision flight simulation turntable, as shown in Figure 6.

When the turntable rotated according to the set trajectory, the original data of the magnetometer was collected by the MCU, and the actual attitude was obtained by the high-precision photoelectric encoder to compare the obtained value with the reference.

The estimated attitude by taking the magnetic measurement as the observation data was compared with the actual one to verify the performance of the proposed AEKF in an off-line way. In order to simulate actual flight conditions, the rotating speed of the turntable is gradually accelerated from 0 r/s to 5 r/s in the space of one second, and the roll angle is maintained at a speed of 5 r/s.

**Figure 7:**
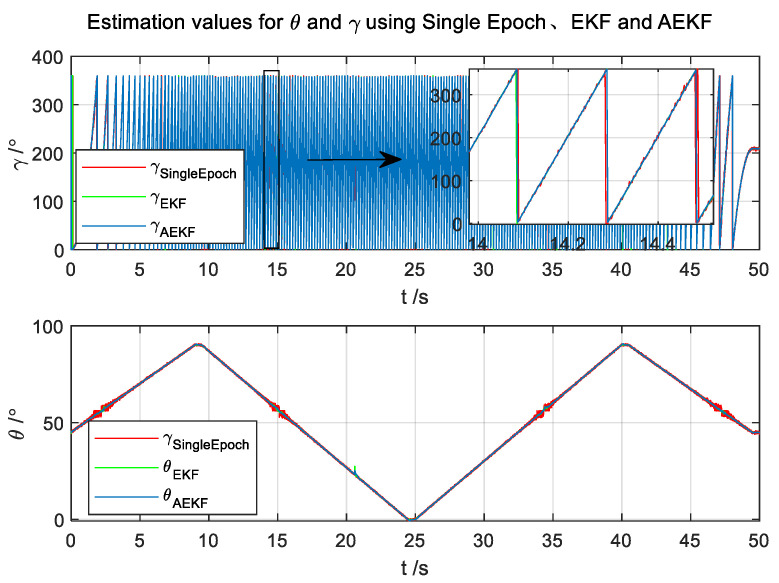
The experimental results.

**Figure 8:**
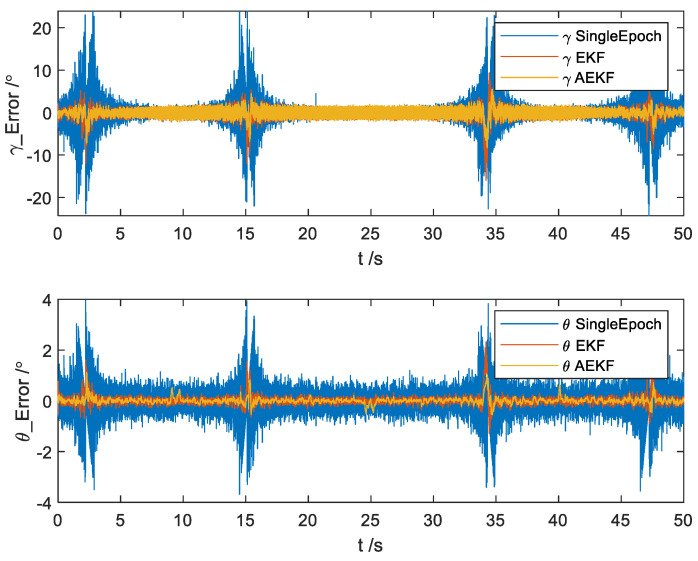
Errors of attitude angle estimation.

**Figure 9:**
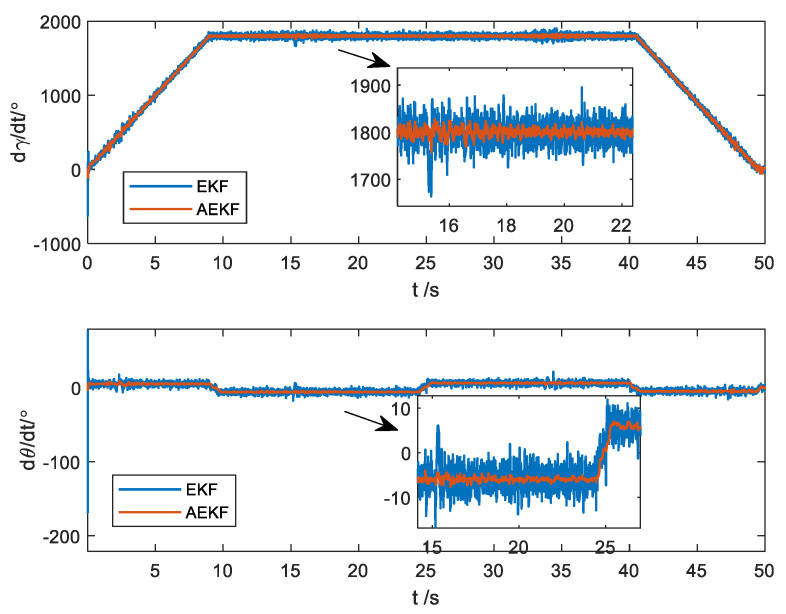
Angular rates estimate.

At the same time, to verify the dual solution problem, the amplitude of the pitch swing was set from 0° to 90°. The pitch angle first varies slowly from 45° to 90°. Then it goes back and forth between 0° and 90° for 48 s; the whole test time is 50 s; the results are shown in Figure 7, Figure 8 and Figure 9.

It can be seen from Figure 7 that the results of the semi-physical experiments, similar to simulation experiments, exhibit a binary ambiguity issue in the pitch and roll angles obtained through the Single-Epoch attitude method. However, our proposed approach effectively mitigates this issue.

It can be seen from Figure 8 that there is a significantly reduced standard deviation in attitude angle errors when employing the AEKF algorithm compared to the traditional Single-Epoch method. Furthermore, the standard deviation is consistently lower than that obtained from the EKF algorithm throughout the entire process.

It can be seen from Figure 9 that the standard deviation of attitude angular rates generated by the AEKF algorithm is smaller than those acquired from the EKF algorithm across the entire process. Notably, the EKF algorithm computes attitude angular rates approximately three times greater than those produced by the AEKF. This result is largely consistent with the simulation findings.

## 4. Conclusions

Obtaining accurate attitude measurements of spinning projectiles is of paramount importance for guided ammunition applications. Attitude measurement systems based on geomagnetism have the advantages of small space occupation, fast response speed, good anti-high overload performance, no accumulated error over time, and so on, making them suitable for projectile operation in environments of overload and high-dynamic flight.

In this article, an estimation algorithm based on AEKF to estimate the pitch and roll angle, the corresponding angular rate, and the corresponding angular acceleration by using only magnetic field information provided by a triaxial magnetometer is proposed. This method primarily aims to eliminate the dual solution problem inherent in traditional Single-Epoch algorithms and provides better mitigation against the impact of magnetic disturbance on attitude solutions while simultaneously providing attitude angular rate information.

Experiments suggest that the proposed method can effectively reduce measurement errors, lower the complexity of the algorithm, and enhance the real-time performance of the pitch and roll angles of spinning projectiles. The standard deviation of the pitch angle and the roll angle, compared with the traditional Single-Epoch attitude algorithm, is improved by four times. The standard deviation of the attitude angular rate is significantly improved compared to the EKF. In addition, the proposed method has many potential applications involving attitude measurement and geomagnetic navigation.

## Figures and Tables

**Table 1:** The standard deviation of attitude angle errors of the Single-Epoch, EKF, and AEKF.

	Contrast Region	Single Epoch (°)	EKF (°)	AEKF (°)
Roll angle	Entire process	1.3671	0.3226	0.2482
	The Binarization region	3.3888	0.7229	0.5640
	Magnetic disturbance region	1.0983	0.4257	0.1899
Pitch angle	Entire process	0.5990	0.1424	0.1137
	The Binarization region	1.2598	0.2534	0.1952
	Magnetic disturbance region	0.8392	0.1677	0.0553

**Table 2:** The standard deviation of attitude angular rate errors of the EKF and AEKF algorithms.

	Contrast Region	EKF (°/s)	AEKF (°/s)
Roll angle	Entire process	0.2430	0.1008
	Magnetic disturbance region	0.5804	0.1094
Pitch angle	Entire process	0.0526	0.0197
	Magnetic disturbance region	0.0447	0.0160

## Data Availability

Not applicable.
